# A novel prognostic signature based on four glycolysis‐related genes predicts survival and clinical risk of hepatocellular carcinoma

**DOI:** 10.1002/jcla.24005

**Published:** 2021-09-15

**Authors:** Zhihong Chen, Yiping Zou, Yuanpeng Zhang, Zhenrong Chen, Fan Wu, Ning Shi, Haosheng Jin

**Affiliations:** ^1^ Medical College of Shantou University Shantou China; ^2^ Department of General Surgery Guangdong Provincial People's Hospital Guangdong Academy of Medical Sciences Guangzhou China

**Keywords:** bioinformatic analysis, glycolysis, hepatocellular carcinoma

## Abstract

**Background:**

Hepatocellular carcinoma (HCC) is the most common cancer with limited cure and poor survival. In our study, a bioinformatic analysis was conducted to investigate the role of glycolysis in the pathogenesis and progression of HCC.

**Methods:**

Single‐sample gene set enrichment analysis (ssGESA) was used to calculate enrichment scores for each sample in TCGA‐LIHC and GEO14520 according to the glycolysis gene set. Weighted gene co‐expression network analysis identified a gene module closely related to glycolysis, and their function was investigated. Prognostic biomarkers were screened from these genes. Cox proportional hazard model and least absolute shrinkage and selection operator regression were used to construct the prognostic signature. Kaplan–Meier (KM) and receiver operating characteristic (ROC) curve analyses evaluated the prediction performance of the prognostic signature in TCGA‐LIHC and ICGC‐LIRI‐JP. Combination analysis data of clinical features and prognostic signature constructed a nomogram. Area under ROC curves and decision curve analysis were used to compare the nomogram and its components.

**Results:**

The glycolysis pathway was upregulated in HCC and was unfavorable for survival. The determined gene module was mainly enriched in cell proliferation. A prognostic signature (CDCA8, RAB5IF, SAP30, and UCK2) was developed and validated. KM and ROC curves showed a considerable predictive effect. The risk score derived from the signature was an independent prognostic factor. The nomogram increased prediction efficiency by combining risk signature and TNM stage and performed better than component factors in net benefit.

**Conclusion:**

The gene signature may contribute to individual risk estimation, survival prognosis, and clinical management.

## Graphical abstract

In the present study, we identified the genes that might interact with the glycolysis pathway, screened the dysregulated genes with prognostic effect and then constructed a novel nomogram integrated by these biomarkers and clinicopathological characteristics to assist in risk stratification and survival prognosis. We observed that the screened genes were not only related to the survival prognosis, but also related to the tumor proliferation and growth, which could be derived from the results of bioinformatics analysis. (1) The glycolysis pathway was upregulated in HCC and correlated with prognosis of HCC. (2) The signature based on 4 glycolysis related genes could effectively perform risk stratification and survival prediction in patients with HCC and 4 genes might be promising biomarkers and potential therapeutic targets in HCC. (3) The nomogram might contribute to individual risk estimation, survival prognosis and clinical management.

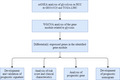

## 1 INTRODUCTION

Liver cancer is the sixth most common cancer and the fourth leading cause of cancer‐related deaths worldwide. Hepatocellular carcinoma (HCC) accounts for 75%–85% of these cases.^1^ Cancer prevention and treatment advances have reduced the overall incidence and mortality rates in recent years. However, liver cancer is gradually increasing and has aggravated the global disease burden.^2^ The main risk factors for HCC include chronic hepatitis B or hepatitis C virus infection, aflatoxin exposure, alcohol abuse, smoking, obesity, and others.^3^ Patients with HCC are usually diagnosed at intermediate or advanced stages due to occult onset and atypical symptoms. Curative treatment methods, including surgical resection, liver transplantation, and locoregional ablation, are unable to achieve the desired effect.^4^, ^5^ Radiotherapy, chemotherapy, targeted therapy, and immunotherapy are integrated into the comprehensive treatment of HCC, which improves the survival prognosis of patients with intermediate and advanced HCC.^6^, ^7^

However, tumor heterogeneity may result in various therapeutic responses and survival outcomes. Even though the histological grade and tumor stage of HCC are the same, biological behavior differences caused by molecular and genetic diversity affect the therapeutic effect.^8^, ^9^ Increasing evidence indicates that energy metabolism reprogramming is important in the initiation and progression of HCC; especially, the shift of aerobic glycolysis from oxidative phosphorylation, which is first found in HCC, is a hallmark of liver cancer and is responsible for the regulation of proliferation, immune evasion, invasion metastasis, angiogenesis, and drug resistance in HCC.^10^, ^11^, ^12^ Aerobic glycolysis can rapidly produce sufficient adenosine triphosphate to meet the energy requirements of tumor growth and proliferation under hypoxic conditions. The metabolic intermediates of glycolysis can serve as raw materials for the synthesis of proteins, lipids, and nucleic acids. The production and accumulation of lactate and hydrogen ions during glycolysis lead to the acidification of the extracellular microenvironment, which can induce apoptosis of peripheral normal cells and produce an immunosuppressive effect on effector immune cells, prompting the invasion and metastasis of tumor cells.^13^ Glycolysis is regulated by glycolytic enzymes and by transcription factors that include c‐MYC, hypoxia inducible factor‐1 (HIF‐1), and p53, which are related to the expression and alteration of multiple oncogenes.^14^ Consequently, antiglycolytic therapy might inhibit tumor proliferation and kill tumor cells. Glycolytic rate‐limiting enzymes and HIFs are ideal targets for HCC.^15^ Key glycolytic enzymes, such as hexokinase 2 (HK2), phosphofructokinase (PFK), and pyruvate kinase 2 (PKM2), are reported tumor markers.^16^, ^17^ Their expression and activity can affect the glycolysis of tumors, in turn affecting the proliferation of tumors. Moreover, inhibition of glycolysis can increase the sensitivity of advanced HCC to sorafenib in cell and animal models.^18^ In addition, 18F‐fluorodeoxyglucose positron emission tomography/computed tomography (18FDG‐PET/CT) enables the location, diagnosis, and follow‐up of HCC based on glycolysis.^19^

The risk signature for the prognosis of HCC involving glycolysis‐related genes has been investigated previously.^20^, ^21^, ^22^ Nevertheless, the function of genes that might interact with glycolysis pathway in HCC has not been explored. Here, we analyzed the role of the glycolysis pathway in HCC tumorigenesis, screened the genes that might interact with glycolysis pathway, and assessed their important prognostic value. The identified genes enabled the construction of a highly efficient prognostic model to predict the survival and clinical risk of HCC. In addition, a nomogram that combined gene signatures and clinical characteristics displayed a higher prediction performance. The results reveal some novel promising targets for HCC that appear to be very important for prognosis evaluation, risk stratification, and reasonable treatment options.

## 2 MATERIALS AND METHODS

### 2.1 Data collection and processing

The transcriptome data of TCGA‐LIHC and the corresponding clinical information were downloaded from The Cancer Genome Atlas (TCGA (http://cancergenome.nih.gov). The transcriptomic gene expression profiles of ICGC‐LIRI‐JP and GSE14520, and corresponding clinical data were obtained from International Cancer Genome Consortium (ICGC, https://dcc.icgc.org/) and Gene Expression Omnibus (GEO, https://www.ncbi.nlm.nih.gov/geo/). Due to the unknown identity of patients from the above cohorts, informed consent was waived. Patients with incomplete survival information and a survival period of less than 30 days were excluded. Their detailed information is provided in Table 1. Two hundred genes encoding proteins involved in glycolysis and gluconeogenesis were extracted from the gene set (HALLMARK_GLYCOLYSIS) retrieved from the Molecular Signatures Database of GSEA (https://www.gsea‐msigdb.org/gsea/index.jsp).

**TABLE 1:** Clinicopathological information of the patients in TCGA‐LIHC cohort and ICGC‐LIRI‐JP cohort

	Survival status		Total (*N* = 348)
	Alive (*N* = 223)	Dead (*N* = 125)	
Age (years old)			
Mean (*SD*)	58.4 (12.9)	61.2 (13.7)	59.4 (13.2)
Median [Min, Max]	60.0 [16.0, 81.0]	64.0 [18.0, 90.0]	61.0 [16.0, 90.0]
Gender			
Female	61	49	110
Male	162	76	238
Histological grade			
G1	36	17	53
G2	104	60	164
G3	74	39	113
G4	8	5	13
AJCC TNM stage			
Stage I	126	39	165
Stage II	54	24	78
Stage III	36	45	81
Stage IV	0	3	3
T stage			
T1	129	43	172
T2	57	28	85
T3	32	43	75
T4	3	10	13
M stage			
M0	170	80	250
M1	0	3	3
N stage			
N0	166	78	244
N1	1	2	3

	Survival status		Total (*N* = 256)
	Alive (*N* = 214)	Dead (*N* = 42)	
Age (years old)			
Mean (*SD*)	67.5 (10.2)	66.9 (9.57)	67.4 (10.1)
Median [Min, Max]	69.0 [31.0, 89.0]	68.5 [37.0, 83.0]	69.0 [31.0, 89.0]
Gender			
Female	51	17	68
Male	163	25	188
TNM stage			
Stage I	39	1	40
Stage II	99	18	117
Stage III	63	13	76
Stage IV	13	10	23

Abbreviations: AJCC, American Joint Committee on Cancer; G1, high differentiation; G2, medium differentiation; G3, low differentiation; G4, undifferentiated; LCSGJ, Liver Cancer Study Group of Japan.

### 2.2 Single‐sample gene set enrichment analysis (ssGSEA) and weighted gene co‐expression network analysis (WGCNA)

ssGSEA was applied to calculate the enrichment score that represented the absolute enrichment degree of a gene set in each sample by an empirical cumulative distribution function based on the given gene expression profile. Glycolysis ssGSEA scores of 424 samples from TCGA‐LIHC and 445 samples from GSE14520 were derived using the Gene Set Variation Analysis (GSVA) package. The scores were compared in different tissue types and survival statuses using the limma package in R.^23^ Gene expression profiles of 374 tumor samples and 50 normal samples were used for gene set enrichment analysis (GSEA) on the gene set (HALLMARK_GLYCOLYSIS) with 1000 permutations using GSEA version 4.1.0.^24^ WGCNA was used to identify highly correlated gene modules and to analyze the association between the gene modules and phenotype information via the WGCNA package in R. The gene module with a highly significant positive correlation with glycolysis pathway genes was selected for further analysis.^25^

### 2.3 Development and validation of the novel prognostic signature

Differential gene expression analysis of the identified gene module between 374 HCC tumor samples and 50 adjacent non‐tumor samples in the TCGA‐LIHC cohort was performed. Abnormally expressed genes were identified using the limma package in R software. The threshold was a false discovery rate (FDR) < 0.05 and log2 |fold change (FC) | > 1.^26^ The differentially expressed genes (DEGs) were extracted and analyzed with the Gene Ontology (GO) term annotation and Kyoto Encyclopedia of Genes and Genomes (KEGG) pathway functional enrichment analysis to investigate their underlying biological mechanism using clusterProfiler package in R software.^27^

The Cox proportional hazards model and least absolute shrinkage and selection operator (LASSO) regression were used to identify prognostic DEGs and construct a prognostic signature associated with overall survival (OS) of patients with HCC.^28^ Individual risk scores were calculated using the generated coefficients and their corresponding gene expression. In accordance with the median value of risk scores, we divided the cohort into high‐risk and low‐risk groups and used Kaplan–Meier (KM) survival curves and time‐dependent receiver operating characteristic (ROC) curves to assess discrimination ability and prediction accuracy of the prognostic signature. We used the LIRI‐JP cohort in the ICGC database as an external validation cohort to verify the prognostic effect of the prognostic signature. The same formula was used to calculate individual risk scores. For this, patients were classified into the high‐risk and low‐risk groups according to the median value of the risk scores. KM survival curves and time‐dependent ROC curves were obtained, as described above. Principal component analysis (PCA) and t‐distributed stochastic neighbor embedding (t‐SNE) were used to detect the clustering ability of the prognostic signature. Subgroup survival analyses of age, gender, histological grade, AJCC TNM stage, T stage, N stage, and M stage were performed according to the median risk scores of each subgroup. The Chi‐square test was used to evaluate the relationship between different risk levels and diverse clinical characteristics. The Wilcoxon rank‐sum test was used to compare risk scores in different clinical subgroups. Univariate and multivariate Cox regression analyses were used to confirm the independent prognostic impact of risk scores and several clinical factors on patient outcomes. Gene Expression Profiling Interactive Analysis (GEPIA, http://gepia.cancer‐pku.cn/) was applied to demonstrate the prognostic effect of the screened genes.^29^ The cBioPortal for Cancer Genomics (http://www.cbioportal.org/) was employed to illustrate the mutation state of the selected genes.^30^

### 2.4 Development of the nomogram based on prognostic signature and clinical characteristics

The clinical characteristics (*p* < 0.05) were considered as candidate prognostic factors by univariate Cox regression analysis and were entered into the stepwise multivariate Cox regression analysis with the aforementioned prognostic signature. A nomogram combining prognostic signatures and clinical features was developed. We analyzed the prediction, discrimination, and calibration abilities of the nomogram using the concordance index (C‐index), time‐dependent ROC curves, KM survival curves, and calibration curves. The area under the curve (AUC) of the ROC curves of the nomogram and other prognostic factors, such as the AJCC TNM stage and four‐gene based prognostic signature, were assessed and compared at different time points over the follow‐up period using time‐dependent AUC curves. Decision curve analysis (DCA) was used to examine the benefits of different prediction models.

### 2.5 Statistical analysis

R version 4.0.3 software (https://cran.r‐project.org/) was the main tool used to conduct the statistical analysis. A *p*‐value of <0.05 was considered statistically significant.

## 3 RESULTS

### 3.1 Role of the glycolysis pathway in HCC

**FIGURE 1:**
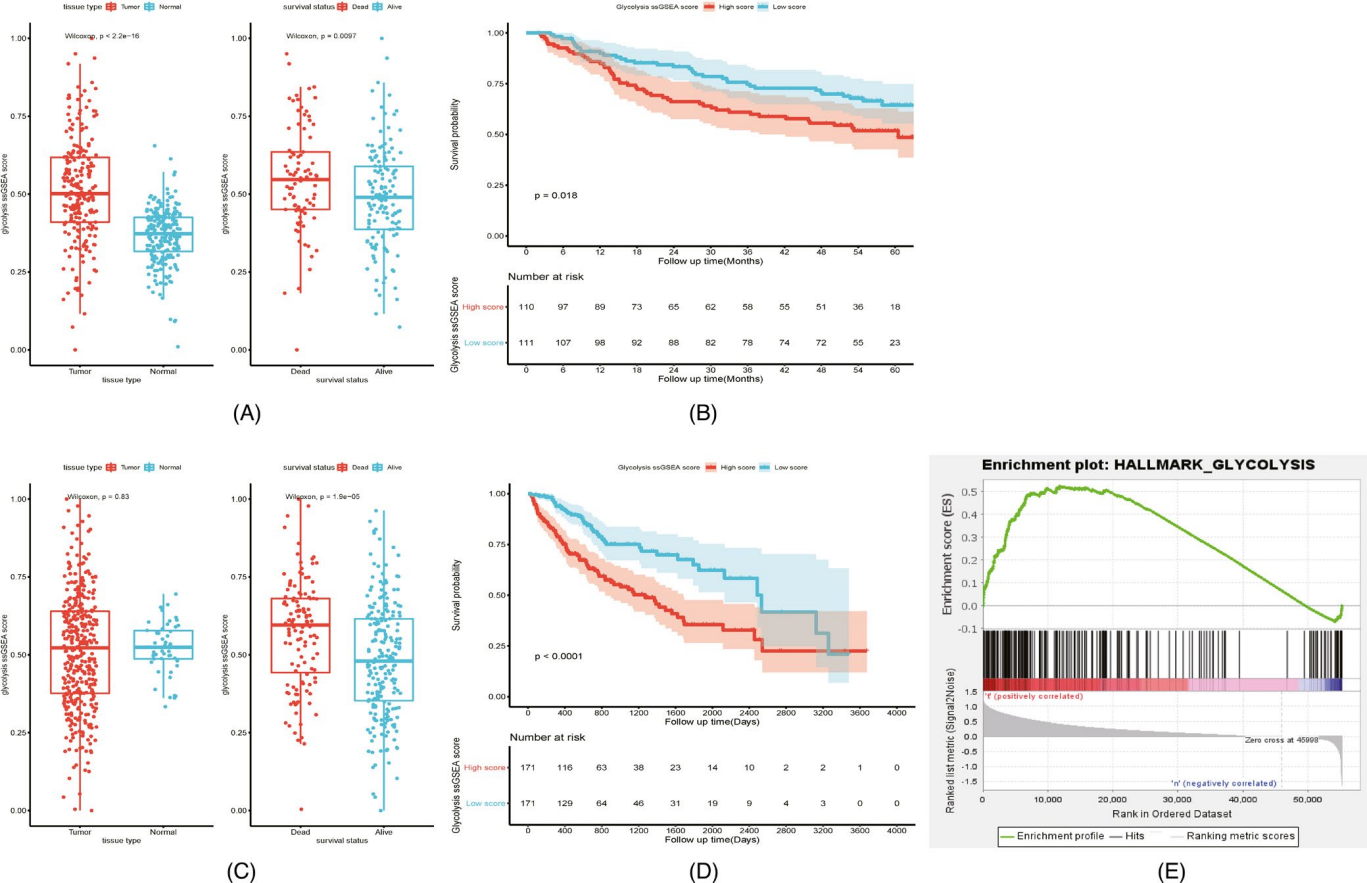
ssGSEA analysis of glycolysis pathway (HALLMARK_GLYCOLYSIS) in HCC. Comparison of glycolysis ssGSEA scores among different subgroups in GEO14520 (A). Influence of glycolysis ssGSEA score level on survival prognosis in GEO14520 (B). Comparison of glycolysis ssGSEA scores among different subgroups in TCGA‐LIHC (C). Influence of glycolysis ssGSEA score level on survival prognosis in TCGA‐LIHC (D). Gene set enrichment plots of HALLMARK_GLYCOLYSIS in TCGA‐LIHC (E)

Glycolysis ssGSEA scores were calculated for each sample in GSE14520 and TCGA‐LIHC to represent the glycolytic activity using the ssGSEA method from the GSVA package, according to the gene set HALLMARK_GLYCOLYSIS. In GSE14520, it was observed that the glycolysis ssGSEA score was significantly higher in tumor tissues than in matched normal tissues (*p* < 0.05), and the glycolysis ssGSEA score of the dead patients was significantly higher than that of the surviving patients (*p* < 0.05) (Figure 1A). Compared to the patients with lower glycolysis ssGSEA scores, the patients with higher glycolysis ssGSEA scores had worse outcomes (*p* < 0.05) (Figure 1B). In TCGA‐LIHC, although no significant difference was found in the comparison of glycolysis ssGSEA scores between tumor tissues and adjacent normal tissues (*p* = 0.83), glycolysis ssGSEA scores of the patients with different survival outcomes were distinctly different, similar to the results of GSE14520 (*p* < 0.05) (Figure 1C). The results of KM survival analysis indicated that the patients with higher glycolysis ssGSEA scores had a shorter survival period than those with lower glycolysis ssGSEA scores (*p* < 0.05) (Figure 1D). The results of GSEA analysis in the TCGA‐LIHC cohort showed that the normalized enrichment score of gene set HALLMARK_GLYCOLYSIS was more than 1 and both the NOM *p*‐value and FDR *q*‐value were <0.05, indicating that the biological behaviors of glycolysis and gluconeogenesis were more active in tumor tissues than in normal tissues in HCC, and it might promote the tumorigenesis and development (Figure 1E).

### 3.2 Novel prognostic signature based on four genes

**FIGURE 2:**
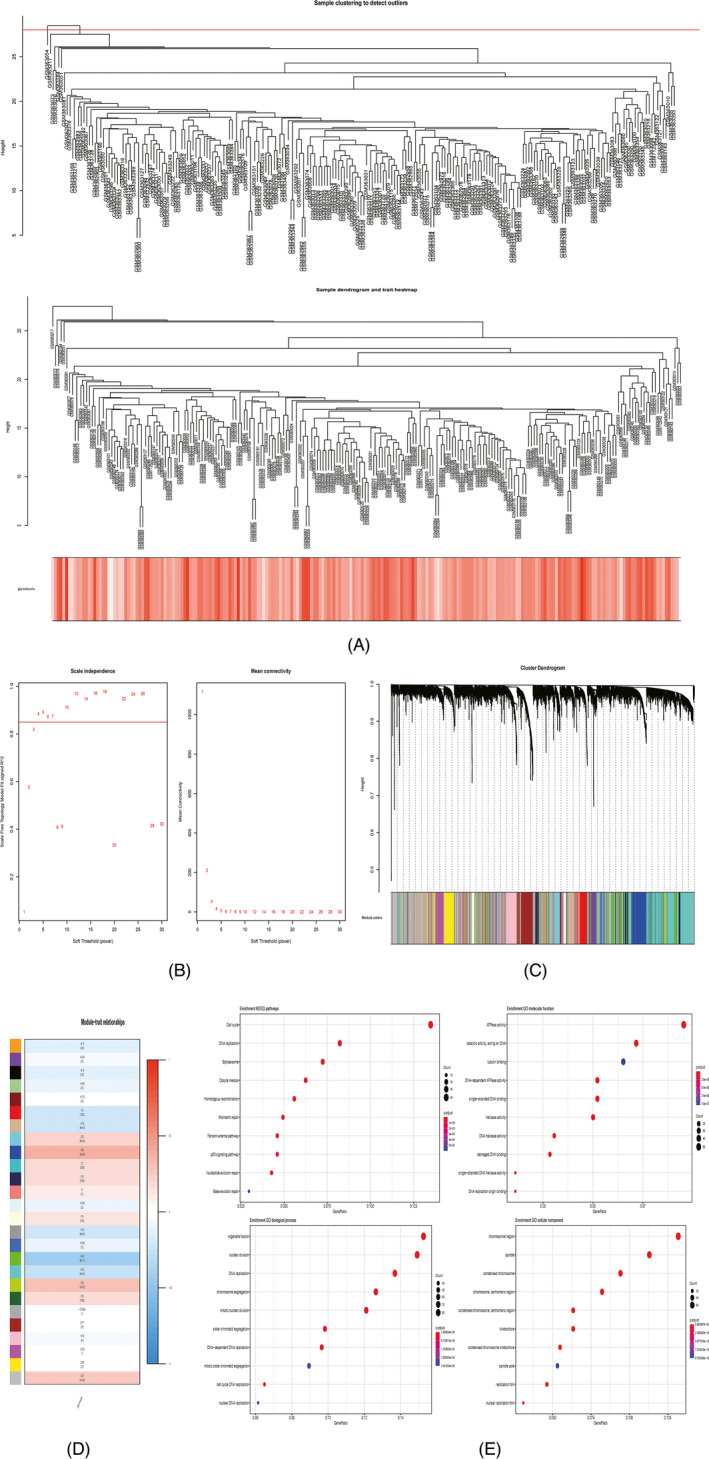
WGCNA analysis of gene expression profiles in HCC. One outlier was detected by sample clustering, sample dendrogram, and trait heatmap (A). Soft threshold equaled 4 when scale independence tended to the maximum and mean connectivity tended to the minimum (B). Hierarchical clustering tree revealing 26 gene modules (C). Correlation between the 26 gene modules and glycolysis ssGSEA scores (D). Gene enrichment analysis of the blue gene module (E)

WGCNA was implemented to construct a gene co‐expression network and to identify a highly synergistic gene set based on weighted gene expression correlation. After excluding one discrete sample (GSM363054) and setting the optimal soft threshold to 4, hierarchical clustering analysis was performed. The clustering results were segmented to obtain many highly positively correlated gene modules, represented by different color branches of the clustering tree (Figure 2A‐C). The correlation between gene modules and glycolysis ssGSEA score was calculated and compared. The blue gene module was determined to have the most positive correlation with glycolysis ssGSEA scores (Cor = 0.39, *p* < 0.05) (Figure 2D). A total of 614 genes in the blue gene module were extracted to conduct GO term annotation and KEGG pathway analysis. The ten most significantly enriched KEGG pathways of these genes were cell cycle, DNA replication, spliceosome, oocyte meiosis, homologous recombination, mismatch repair, Fanconi anemia pathway, p53 signaling pathway, nucleotide excision repair, and base excision repair. The results of GO term analysis showed that these genes were mainly enriched in biological processes, cellular components, and molecular functions associated with cell proliferation and division (Figure 2E).

**FIGURE 3:**
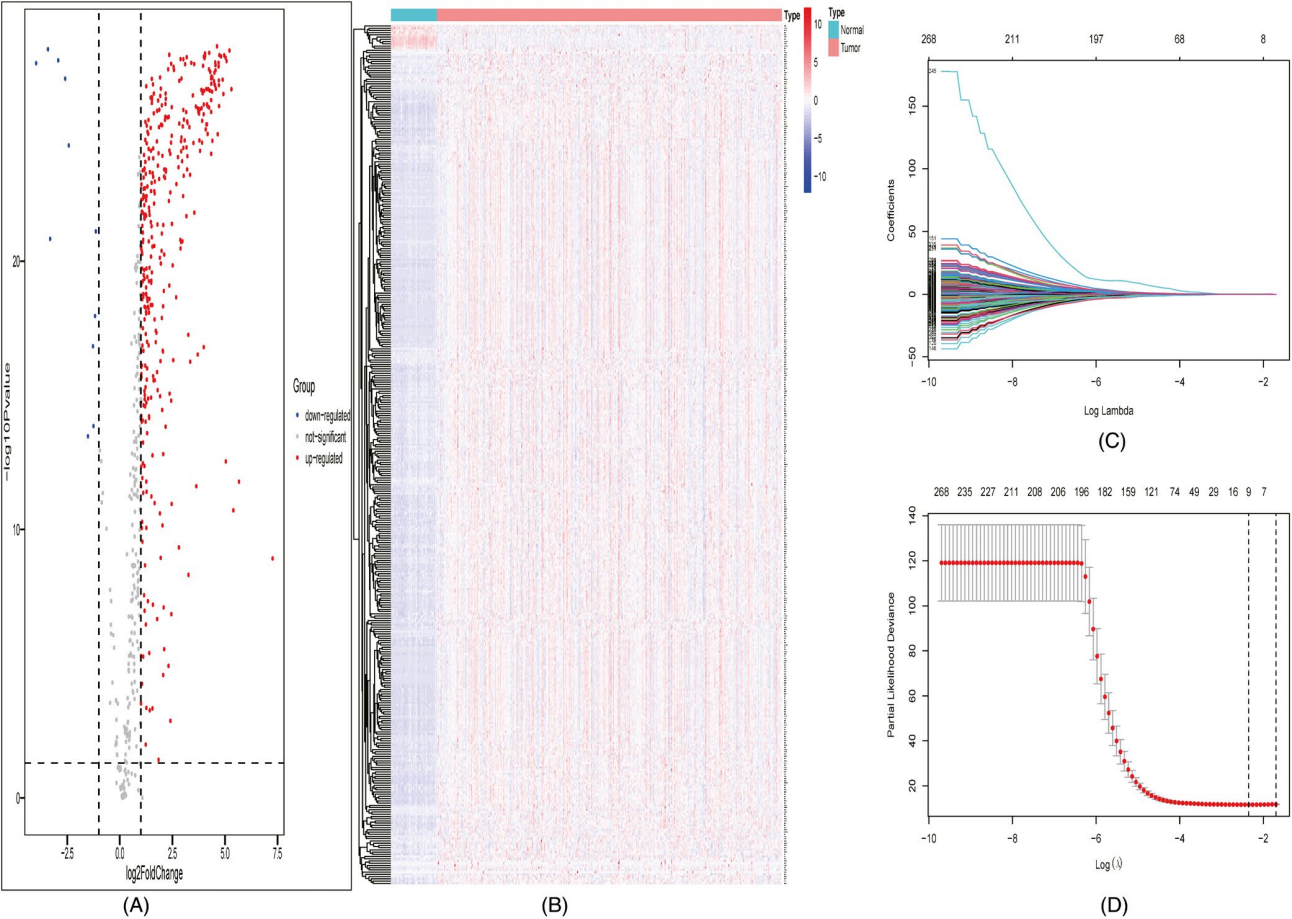
DEG and LASSO regression analyses. Volcano plot of the DEGs (A). Heatmap of the DEGs (B). Filtration of variables in LASSO regression (C). 9 DEGs were obtained from LASSO regression (D)

After DEG analysis of 614 genes in the cohort of TCGA‐LIHC, we identified 375 upregulated genes and 11 downregulated genes between tumor samples and adjacent normal samples (Figure 3A,B). A total of 339 DEGs were screened after univariate Cox regression analysis (*p *< 0.05). Of these, nine (C5orf30, CDCA8, GTPBP4, NEIL3, PPM1G, PSRC1, RAB5IF, SAP30, and UCK2) were chosen as prognostic DEGs. The number of candidate genes was reduced by LASSO regression analysis (Figure 3C,D). Multivariate Cox stepwise regression analysis was used to construct the prognostic signature via the analysis of these nine DEGs. The regression coefficient and gene expression of the four enrolled DEGs (CDCA8, RAB5IF, SAP30, and UCK2) were calculated. Four DEGs were integrated into a formula based on the product of gene expression and regression coefficient. The individual risk score was calculated as follows: Risk score = (expression of CDCA8 * 0.316) + (expression of RAB5IF * 0.378) + (Expression of SAP30 * 0.265) + (Expression of UCK2 * 0.334) (Table 2). All four DEGs were upregulated in HCC and high expression levels were unfavorable indicators for OS of the patients.

**TABLE 2:** Stepwise multivariate Cox regression analysis of survival prognostic genes

Id	Expression	Coefficient	HR	95% CI	*p* value
CDCA8	Up‐regulated	0.316	1.372	1.065–1.767	0.014*
RAB5IF	Up‐regulated	0.378	1.459	1.105–1.928	0.008**
SAP30	Up‐regulated	0.265	1.304	0.954–1.783	0.096
UCK2	Up‐regulated	0.334	1.396	1.014–1.923	0.041*

Abbreviations: CI, confidence interval; HR, hazard ratio.

*p < 0.05, **p < 0.01, ***p < 0.001.

**FIGURE 4:**
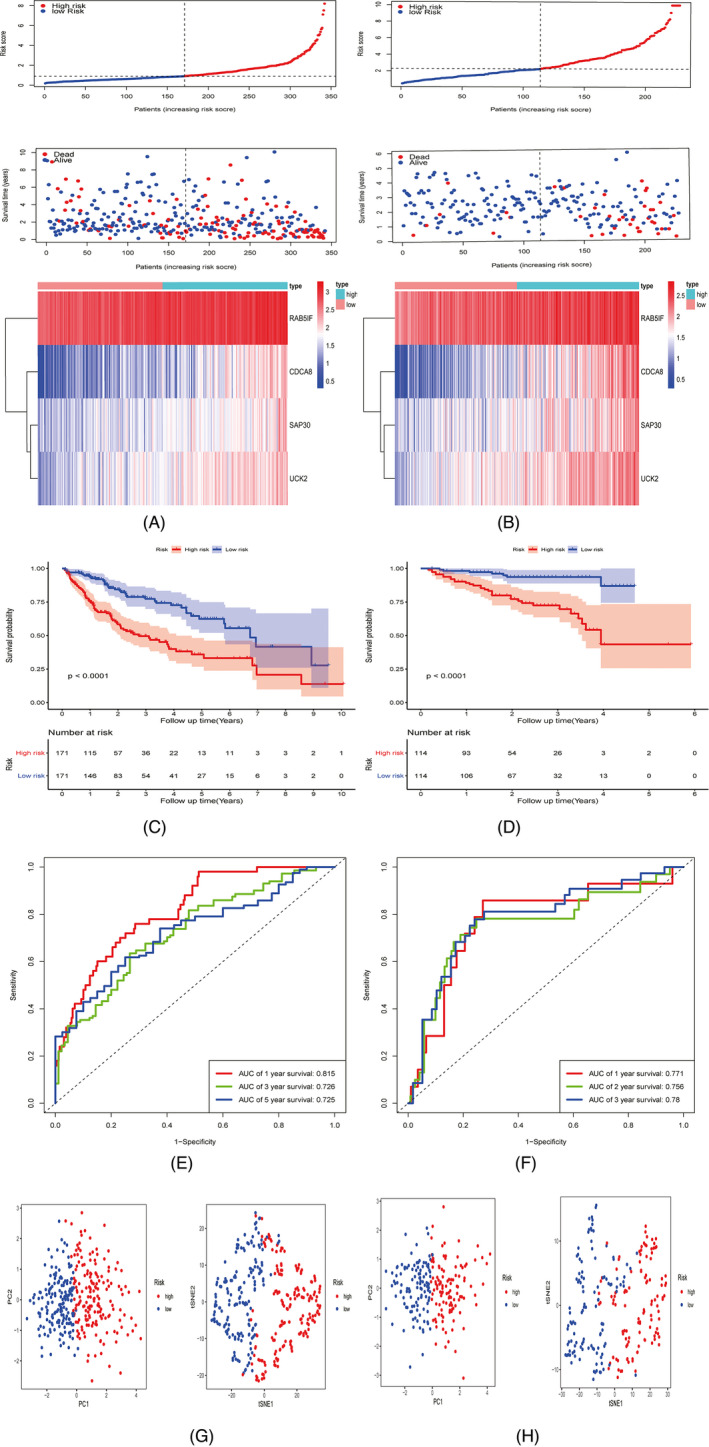
Development and validation of prognostic signature based on four genes (CDCA8, RAB5IF, SAP30, and UCK2) in TCGA‐LIHC and ICGC‐LIRI‐JP cohorts. The risk and survival distribution of the patients, and the heatmap of the four genes in TCGA‐LIHC and ICGC‐LIRI‐JP cohorts (A‐B). KM curve revealing the significantly shorter OS in the high‐risk group compared with the low‐risk group in TCGA‐LIHC and ICGC‐LIRI‐JP cohorts (C‐D). ROC curve of the risk signature in TCGA‐LIHC and ICGC‐LIRI‐JP cohorts (E‐F). PCA and t‐SNE analyses of low‐ and high‐risk groups in TCGA‐LIHC and ICGC‐LIRI‐JP cohorts (G‐H)

Taking the median value of risk scores as the cutoff value, the cohort of TCGA‐LIHC was divided into the high‐risk and low‐risk groups. The distribution of risk scores, survival status, and expression of the four genes are displayed in Figure 4A. With the increase in risk score, more patients tended to be in the state of death. The heatmap indicated that four genes were overexpressed in the high‐risk group compared to the low‐risk group. KM curves demonstrated that the prognosis of the high‐risk group was significantly poorer than that of the low‐risk group in OS (*p* < 0.05) (Figure 4C). Time‐dependent ROC curves were used to detect the predictive effect of the risk signature on the OS of patients. The AUC value was used to represent the prediction ability. The AUC values at 1‐, 3‐, and 5‐years were 0.815, 0.726, and 0.725, respectively (Figure 4E). The ICGC‐LIRI‐JP cohort served as the validation cohort for the risk signature. According to the median value of the overall risk scores, the patients from the ICGC‐LIRI‐JP cohort were classified into the high‐risk and low‐risk groups. The distribution of risk scores, survival status, and expression of the four genes are shown in Figure 4B. The results were similar to those of TCGA‐LIHC cohort. Similarly, there were more deaths among those at high risk. The heatmap revealed that the expression comparison results of four genes between high‐ and low‐risk groups were consistent with those in TCGA‐LIHC cohort. KM curves suggested that the high‐risk group had a markedly lower OS rate than the low‐risk group (*p* < 0.05) (Figure 4D). Time‐dependent ROC curves of the ICGC‐LIRI‐JP cohort were plotted. The AUC values at 1‐, 2‐, and 3‐years were 0.771, 0.756, and 0.78, respectively (Figure 4F). PCA and t‐SNE analyses illustrated that the prognostic signature could clearly distinguish the high‐risk and low‐risk groups according to the sample distribution of the two groups (Figure 4G,H).

**FIGURE 5:**
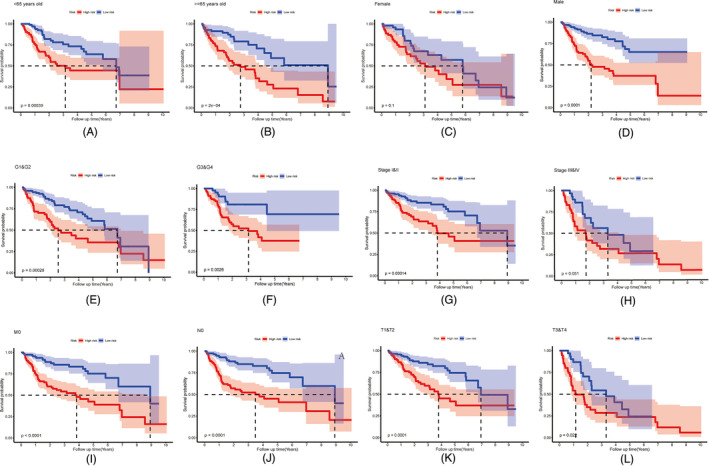
Survival analysis of risk signature in the HCC patients with different clinical features in TCGA‐LIHC, including age (A‐B), gender (C‐D), histological grades (E‐F), AJCC TNM stages (G‐H), M0 stage (I), N0 stage (J) and T stages (K‐L)

**FIGURE 6:**
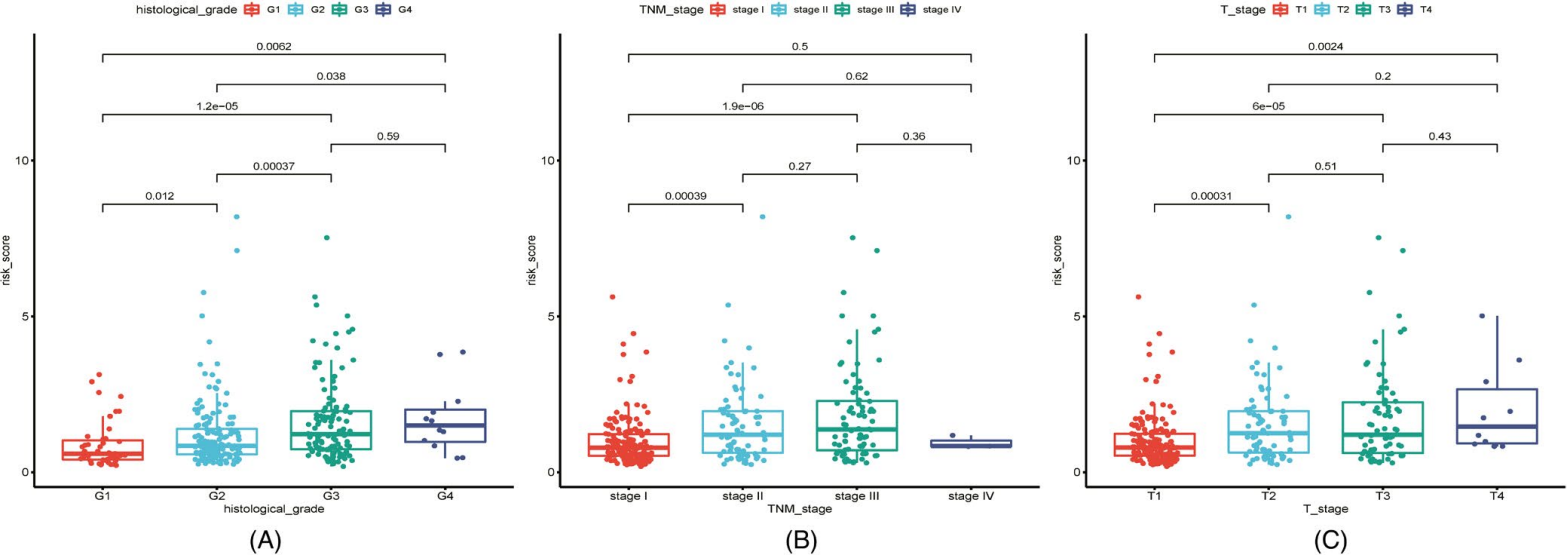
Correlation between the risk scores and HCC patients' histological grades, AJCC TNM stages, and T stages in TCGA‐LIHC (A‐C)

In the cases of age <65 years, age ≥65 years, male sex, TNM stage I‐II, T1‐2, T3‐4, M0, N0, G1‐2, and G3‐4, the KM analysis indicated that the high‐risk group had a significantly poorer OS prognosis than the low‐risk group (all *p* < 0.05). However, the OS of the high‐risk and low‐risk groups in the female subgroup (*p* = 0.1) and TNM stage III‐IV subgroup (*p* = 0.051) did not show significant differences (Figure 5A‐L). Thus, the risk signature might be suitable for risk stratification and survival prediction in most subgroups. The results of the chi‐square test showed that the risk score was related to histological grade, AJCC TNM stage, and T stage (all *p* < 0.05) (Table 3). The results of the Wilcoxon rank‐sum test indicated that the patients with higher risk scores tended to have poorer histological grade, advanced AJCC TNM stage, and higher T stage of HCC (Figure 6A‐C). The results of univariate and multivariate Cox regression analyses in TCGA‐LIHC and ICGC‐LIRI‐JP cohorts suggested that risk scores could act as independent prognostic factors for OS in patients with HCC (*p* < 0.05) (Table 4).

**TABLE 3:** Chi‐square test compared the clinicopathological characteristics of the patients with different risk levels

	Risk		*p* value
	Low (*N* = 171)	High (*N* = 171)	
Age			0.825
<65 years old	101	104	
>=65 years old	70	67	
Gender			0.164
Female	48	61	
Male	123	110	
Histological grade			<0.001***
G1	38	15	
G2	88	73	
G3	40	71	
G4	3	9	
AJCC TNM stage			<0.001***
Stage I	101	60	
Stage II	31	46	
Stage III	28	52	
Stage IV	2	1	
T stage			<0.001***
T1	105	63	
T2	31	53	
T3	28	46	
T4	4	9	
N stage			
N0	116	123	
N1	0	3	
M stage			0.607
M0	116	128	
M1	2	1	

Abbreviations: AJCC, American Joint Committee on Cancer; G1, high differentiation; G2, medium differentiation; G3, low differentiation; G4, undifferentiated.

*p < 0.05, **p < 0.01, ***p < 0.001.

**TABLE 4:** Independent prognostic factor analysis for the overall survival in TCGA‐LIHC cohort and ICGC‐ LIRI‐JP cohort

TCGA‐LIHC	Univariate Cox regression			Multivariate Cox regression		
	HR	95% CI	*p* value	HR	95% CI	*p* value
Age	1.005	0.991–1.020	0.474	1.010	0.994–1.025	0.227
Gender	0.754	0.511–1.113	0.156	0.833	0.553–1.254	0.381
Histological grade	1.118	0.862–1.450	0.400	0.991	0.746–1.316	0.948
AJCC TNM stage	1.806	1.461–2.232	<0.001***	1.270	0.576–2.800	0.554
T stage	1.778	1.454–2.174	<0.001***	1.250	0.592–2.642	0.559
Risk score	1.614	1.444–1.802	<0.001***	1.564	1.376–1.778	<0.001***

ICGC‐LIRI‐JP	Univariate Cox regression			Multivariate Cox regression		
	HR	95% CI	*p* value	HR	95% CI	*p v*alue
Age	1.002	0.970–1.035	0.908	0.995	0.961–1.031	0.799
Gender	0.438	0.230–0.833	0.012*	0.334	0.172–0.652	0.001**
LCSGJ TNM stage	2.171	1.475–3.195	<0.001***	2.127	1.477–3.063	<0.001***
Risk score	1.171	1.093–1.254	<0.001***	1.146	1.063–1.237	<0.001***

Abbreviations: AJCC, American Joint Committee on Cancer; CI, confidence interval; HR, hazard ratio; LCSGJ, Liver Cancer Study Group of Japan.

*p < 0.05, **p < 0.01, *** *p *< 0.001.

**FIGURE 7:**
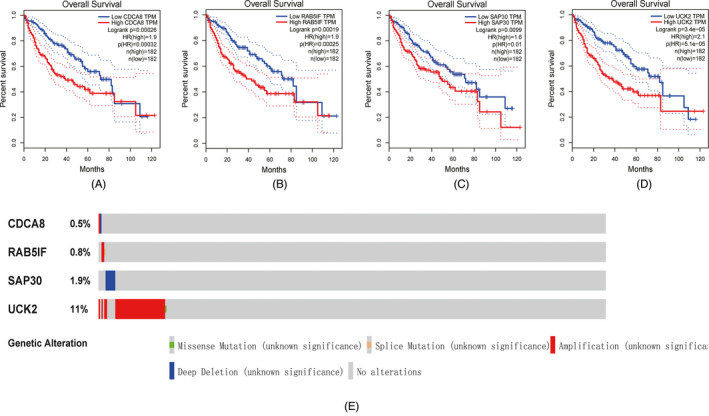
Correlation between the expression of the four identified genes and HCC patients OS in TCGA‐LIHC cohort (A‐D). Mutation of the four genes in TCGA‐LIHC cohort from Firehorse Legacy database (E)

GEPIA results revealed that the expression of all four genes (CDCA8, RAB5IF, SAP30, and UCK2) was related to the OS prognosis of patients with HCC, and patients with higher gene expression levels were more likely to have poorer outcomes (all *p* < 0.05) (Figure 7A‐D). The mutations of four genes in HCC are shown in Figure 6C. UCK2 had the highest mutation rate (11%), especially in amplification. The other three genes had a mutation rate of <2%, with a rate of 0.5% for CDCA8 (Figure 7E).

### 3.3 Nomogram integrating risk signature and clinicopathological features

**FIGURE 8:**
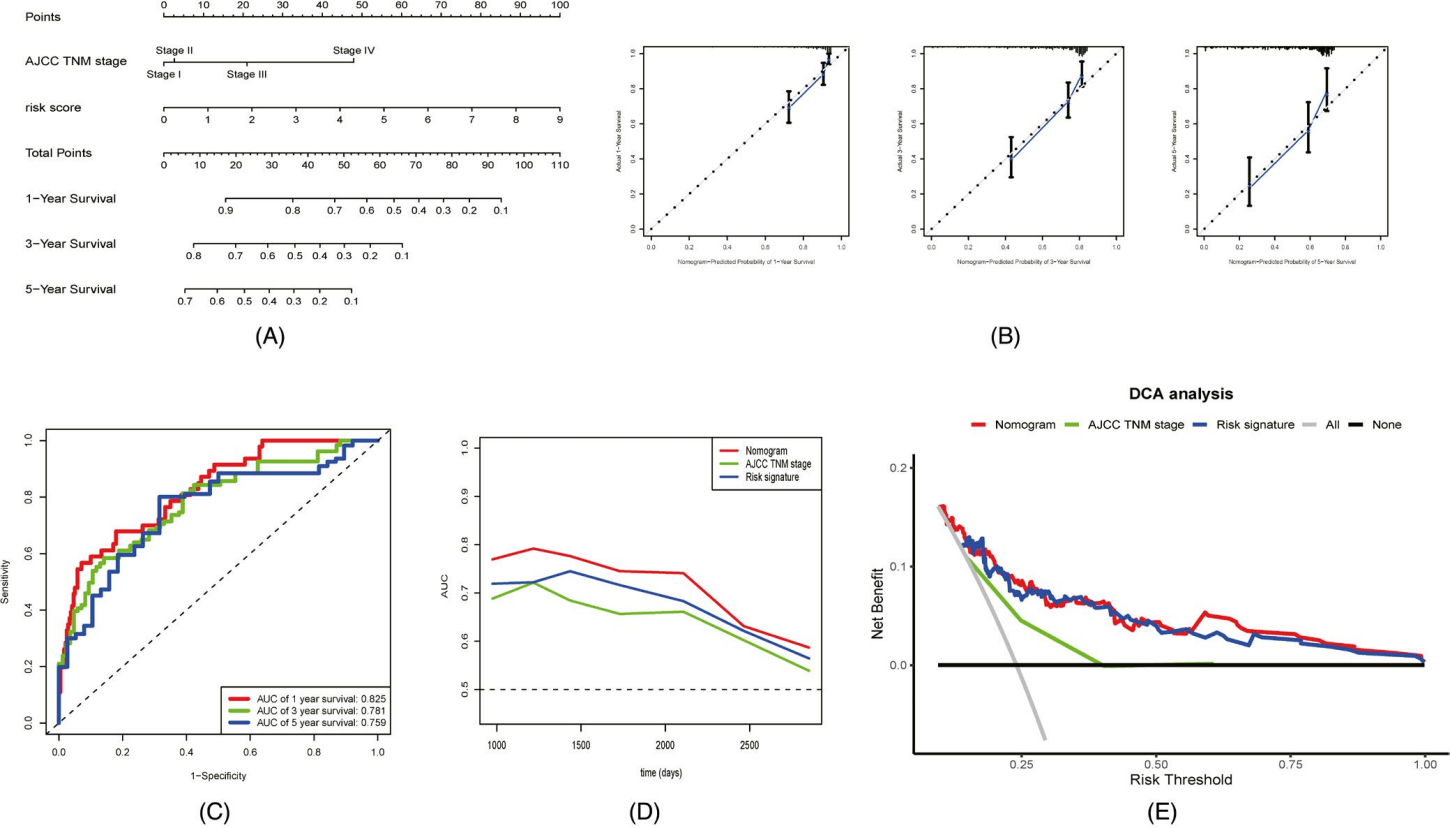
Development of the nomogram consisted of AJCC TNM stage and four‐gene based prognostic signature. Nomogram plot (A). Calibration plots of the nomogram to predict 1‐, 3‐, and 5‐year OS (B). ROC curves of the nomogram to predict 1‐, 3‐, and 5‐year OS (C). Time‐dependent AUC curves of the nomogram and its components (d). DCA analysis of the nomogram and its components (E)

The candidate factors with prognostic value, including AJCC TNM stage and risk scores calculated by the prognostic signature, were screened out by stepwise multivariate Cox regression analysis and were used to construct a highly accurate predictive nomogram (Figure 8A). The nomogram had a C‐index of 0.737 (95% confidence interval [CI]: 0.686–0.788), which was greater than the C‐index of the risk prognostic signature (C‐index, 0.733; 95% CI: 0.682–0.784). The calibration curves showed considerable agreement between the nomogram‐predicted OS and actual OS (Figure 8B). According to the median value of risk scores calculated by the nomogram, the cohort was divided into the high‐risk and low‐risk groups. The results of the KM analysis demonstrated that the prognosis of the low‐risk group was notably better than that of the high‐risk group (*p* < 0.05) (Figure 8C). The nomogram could provide a more accurate survival prediction. ROC analysis suggested that the AUC values at 1, 3, and 5 years for the training cohort were 0.825, 0.781, and 0.759, respectively (Figure 8D). Time‐dependent AUC curves suggested that the predictive power of the nomogram was superior to other predictive models, such as the AJCC TNM stage and four‐gene based prognostic signature at different time points over the follow‐up period. DCA analysis showed that the nomogram could yield superior net benefits compared with an all‐or‐none treatment strategy and other predictive models (Figure 8E).

## 4 DISCUSSIONS

HCC is a common malignant disease with a high incidence and mortality. Most patients with HCC are diagnosed at the terminal stage and have lost the chance to benefit from radical surgery.^31^ Although an increasing number of alternative therapy strategies have been developed and can prolong survival time for patients with unresectable HCC, they are not absolutely effective and some patients still have poor responses.^32^ The reasons why patients respond differently to the same treatment may reflect the heterogeneity of tumors caused by molecular dysregulation and genetic variation.^33^ Therefore, biomarkers related to tumor development and curative effect are worth exploring and could be conducive to early detection, targeted therapy, and personalized management of HCC.^34^, ^35^, ^36^

Glucose metabolism reprogramming, especially aerobic glycolysis, is important in the initiation and progression of HCC in a hypoxic environment, such as growth and proliferation, invasion and metastasis, immunosuppression, and drug resistance.^37^ The results of ssGSEA comparison indicated that glycolysis was more active in HCC and could promote tumor malignant behavior. The regulation of glycolysis in HCC is complex and may be related to genetic mutation and epigenetic modulation of oncogenes and suppressor genes, noncoding RNAs, signaling pathways, glycolytic enzymes, and other factors.^38^ Understanding the mechanism of glycolysis in tumor development is essential to find novel biomarkers and promising therapeutic targets, and to improve diagnosis, treatment, and surveillance of HCC.

In the present study, we constructed co‐expression gene modules related to glycolysis by WGCNA and identified four potential prognostic biomarkers to develop a novel risk signature for HCC. Cell division cycle‐associated 8 (CDCA8), a regulator of mitosis, is overexpressed in bladder cancer, breast cancer, liver cancer, and other tumors and is involved in their growth and development. High CDCA8 expression may be a predictor of poor prognosis in HCC.^39^ Long noncoding RNA RAB5 interacting factor (RAB5IF) is highly expressed in HCC and correlates with the poor prognosis of patients with HCC. RAB5IF can regulate the growth of HCC cells by modulating LGR5 mediated β‐catenin and c‐Myc signaling.^40^ Transcription factor sin3A‐associated protein 30 (SAP30), an indispensable component of the histone deacetylase complex, can regulate the transcription and expression of specific genes, especially tumor suppressor ING1.^41^, ^42^ Uridine‐cytidine kinase 2 (UCK2), which can catalyze the phosphorylation of uridine and cytidine to uridine monophosphate (UMP) and cytidine monophosphate (CMP), is associated with cell proliferation and is a potential unfavorable prognostic predictor for HCC prognosis.^43^ Cytotoxic agents that target UCK2 enzyme activity to induce cancer cell death are currently investigated in some clinical trials.^44^ The gene function and correlation analyses indicated that the genes were involved in glycolytic metabolic pathway and could link glycolysis and tumor growth. Therefore, the four genes with prognostic value might be promising biomarkers and potential therapeutic targets for HCC and are related to glycolysis regulation, tumor cell proliferation, and division in HCC.

Based on the four identified genes, a new risk prognostic signature for HCC was established and validated. The signature was effective for risk estimation and survival prediction. Additionally, the risk scores calculated by the signature were independent prognostic factors for OS and were related to the biological activity of HCC in advanced tumor stage, larger tumor size, and higher histological grade. The patients with higher risk scores might be prone to die and have higher recurrence risk. The nomogram combining the four‐gene based prognostic signature and AJCC TNM stage increased the precision and reliability of the prediction model and obtained a greater net benefit compared with the component factors. The nomogram might be conducive to risk assessment and clinical decision‐making in the diagnosis, treatment, and prognosis of patients with HCC.

This study had several limitations. First, the results lacked the validation of experimental data. Second, the molecular mechanism of the four genes in the glycolysis pathway remains unclear and needs to be further explored in future studies. Finally, the nomogram should be tested by multicenter studies and analyzed by a comprehensive analysis of clinical information to make it more practical in the clinic.

In summary, our study demonstrated the role of glycolysis pathway in HCC. Based on the cell proliferation‐associated genes interacting with glycolysis, the four‐gene prognostic signature was developed and validated. Finally, a novel prognostic nomogram combining the four‐gene prognostic signature and clinicopathological features could enhance prediction efficiency after the evaluation and might contribute to the clinical application of personal diagnosis and treatment.

## CONFLICT OF INTEREST

The authors declare that there is no conflict of interest regarding the publication of this article.

## AUTHOR CONTRIBUTIONS

Haosheng Jin and Ning Shi contributed to the conception and design of the study. The literature was collected by Zhihong Chen and Yiping Zou. Data acquisition, statistical analysis, and chart making were performed by Zhang et al. Zhihong Chen and Yiping Zou wrote the first draft of the manuscript. All authors commented on previous versions of the manuscript. All authors read and approved the final manuscript.

## Data Availability

The data analyzed in this study can be accessed from public databases. Gene expression profiles and corresponding clinical data were downloaded from GEO14520 (https://www.ncbi.nlm.nih.gov/geo/), TCGA‐LIHC (http://cancergenome.nih.gov), and ICGC‐LIRI‐JP (https://dcc.icgc.org/), and the data were analyzed using R software version 4.0.3 (https://www.r‐project.org/). The gene set related to the glycolysis pathway (HALLMARK_GLYCOLYSIS) was downloaded from the Molecular Signatures Database (MSigDB v6.2, http://software.broadinstitute.org/gsea/msigdb). R codes and processing data could be obtained by contacting with the authors.
